# Benefits of robotic-assisted lymphatic microsurgery in deep anatomical planes

**DOI:** 10.1016/j.jpra.2023.07.001

**Published:** 2023-07-19

**Authors:** Andrea Weinzierl, Carlotta Barbon, Epameinondas Gousopoulos, Donata von Reibnitz, Pietro Giovanoli, Lisanne Grünherz, Nicole Lindenblatt

**Affiliations:** Department of Plastic Surgery and Hand Surgery, University Hospital Zurich, Switzerland

**Keywords:** Lymphatic surgery, Microsurgery, Robotic microsurgery, Robotic-assisted microsurgery, Central lymphatic surgery

## Abstract

Micro- and supermicrosurgeries have become standard techniques for lymphatic reconstruction. As increasingly smaller vessels are being targeted, robotic-assisted surgery has emerged as a new approach to push reconstructive limits owing to its ability of motion scaling and providing better accessibility of deep anatomical regions. The precision of the robot is achieved at the expense of operating speed among other variables; therefore, the surgeon must weigh the enhanced dexterity against the additional operating time and cost required for the robotic surgical system itself to ensure optimal resource utilization.

Here we present a case series of 8 patients who underwent robot-assisted lymphatic microsurgery for omental flap transfer to the axilla and lympho-venous anastomosis. The Symani® Surgical System was used with a conventional microscope or 3D exoscope. The use of 3D exoscope provided clear benefits in terms of surgeon positioning. Moreover, access to the recipient vessels near the thoracic wall was significantly improved with the robotic setup. In addition, suture precision was excellent, resulting in patent anastomoses. Operating time for anastomosis was comparable to that for manual anastomosis and demonstrated a steep learning curve.

The benefits of robotic systems in operating fields with good exposure require further evaluation. However, owing to longer instruments, additional stability, dexterity, and motion precision, robotic systems offer a marked advantage for operating in deep anatomical planes and on small structures. A potentially new field for the implementation of robotic surgery is central lymphatic reconstruction. Progress in terms of operating time and cost is crucial, and future research should validate the effectiveness of robotic-assisted surgery in larger clinical studies.

## Introduction

Microsurgery has become a key element in plastic surgery that has enabled successful lymphatic reconstruction. In the last 2 decades, important advances have been made in the implementation of lympho-venous anastomosis (LVA), vascularized lymph node transfer (VLNT), and lymphatic imaging. Lymphatic surgeons have pushed boundaries even further with the rise of supermicrosurgery, which is defined as surgery for structures of <0.8-mm diameter.^1^ Although experienced microsurgeons can suture structures that are <0.5 mm by hand, challenges such as smaller vessel diameters or fragile tissues may be overcome with the application of robotic assistance. However, high-resolution microscopes and smaller suture materials need to be developed in parallel to take advantage of this advanced technology in the future.

Previously, early robotic surgical systems were used for various purposes, such as neurological biopsies^2^ or prostatectomies.^3^ Since then, they have been successfully used to enhance laparoscopic and minimally invasive surgeries for an ever-growing number of indications with the goal of decreasing invasiveness while maintaining effectiveness.^4^ In the field of plastic surgery, robotic surgical systems were first used only in 2007 for microvascular anastomosis of a transverse rectus abdominis flap using the Da Vinci System.^5^ This was mostly due to technical challenges, such as insufficient magnification or mismatched instrument size. However, in recent years, robotic systems specifically developed for microsurgery have been implemented into clinical practice. Currently, 2 robotic systems are available for reconstructive plastic surgery: the MUSA (Microsure, Eindhoven, The Netherlands) and the Symani® Surgical System (Medical Microinstruments, Inc., Wilmington, DE, USA). Owing to motion scaling, these systems optimize the dexterity and precision of the surgeon in microsurgical procedures. Initial results have been encouraging,^6^ but certain drawbacks (including additional costs and longer operating times) remain as limiting factors for their widespread use.^5^^,^^7^^,^^8^ Therefore, a part of the surgeon's responsibility is to weigh these factors against each other for an optimal use of resources. To minimize the socioeconomic burden on the health care system, currently, robotic-assisted surgery may be used primarily in clinical scenarios where it presents a particular advantage over manual surgical techniques.

Given the above background, in this study, we aim to present our experiences in performing robotic-assisted lymphatic reconstructive surgery using the Symani® Surgical System in deep anatomical planes. Moreover, we outline future perspectives for its use in reconstructive lymphatic surgery along with exemplary cases and the relevant literature to better define its role in the current microsurgical practice.

## Methods

Herein, we present a case series of 8 patients treated with VLNT to the axilla and LVA using the Symani® Surgical System (Table 1). The senior author (NL) performed the surgeries for all patients. In the first 6 cases, the robotic system was used in combination with a conventional microscope (PENTERO® 800, Carl Zeiss AG, Oberkochen, Germany), whereas in the last 2 cases, it was used remotely with a 3D exoscope integrated into an optical microscope (KINEVO® 900, Carl Zeiss AG, Oberkochen, Germany). In this study, 2 clinical cases are presented in detail to demonstrate the feasibility of using the robotic system to perform microanastomoses on a very short pedicle within a deep and confined space. Previously, details about the employed robotic system, setup, and technical specifications have been described in detail by our group.^6^^,^^9^ The present study was approved by the Cantonal Ethics Committee of Zurich (BASEC approval number: 2021-02351). Written consent was obtained from the patients for publication of images or videos. The manuscript was prepared in accordance with the STrengthening the Reporting of OBservational studies in Epidemiology guidelines.

**Table 1:** Demographic and clinical characteristics of the 8 patients who received lymph tissue transfer to the axilla using the Symani® Surgical System at our institution

Patient	Sex	Age [years]	Weight [kg]	Height [m]	BMI [kg/m²]	Diagnosis	Operation
**1**	F	20	59	1.73	19.7	Primary lymphedema of the right arm, stage II-III	LTT omentum to axilla, LTT omentum to ellbow, liposuction
**2**	F	62	90	1.64	33.4	Secondary lymphedema of the right arm, stage II-III (postmastectomy)	LTT omentum to axilla, liposuction
**3**	F	55	87	1.68	30.8	Secondary lymphedema of the left arm (postlumpectomy, postaxillary disscetion)	LTT omentum to left axilla, liposuction
**4**	F	51	79	1.67	28.3	Secondary lymphedema of the left arm, stage II-III (postmastectomy, postaxillary dissection)	LTT omentum to axilla, LVA distal forearm, liposuction
**5**	F	60	65	1.6	25.3	Secondary lymphedema of the left arm, stage I (postmastectomy, postaxillary dissection)	LTT omentum to axilla, LVA distal forearm
**6**	F	56	57	1.53	24.3	Secondary lymphedema of the left arm, stage II (postmastectomy, postaxillary dissection)	LTT omentum to axilla, LVA distal forearm, liposuction
**7**	F	50	108	1.59	42.7	Secondary lymphedema of the right arm, stage III (postmastectomy, postaxillary dissection)	LTT omentum to axilla, liposuction
**8**	F	58	70	1.68	24.8	Secondary lymphedema of the right arm, stage II (postlumpectomy, postaxillary dissection)	LTT omentum to right axilla, LVA distal right forearm

LTT = lymphatic tissue transfer; LVA = lympho-venous anastomosis

## Results

All patients operated with the Symani® Surgical System for VLNT to the axilla and LVA were women aged between 20 and 62 years (Table 1). One young woman had primary lymphedema of the arm, whereas all other patients had secondary lymphedema of the arm due to breast cancer treatment. Skin incisions within the axilla were kept as short as possible, usually between 5 and 8 cm. Scar tissue was meticulously released, and the axillary vein was exposed. All omental flaps were connected to the branches of the thoracodorsal vessels, approximately 3 cm from their origin from the axillary vessels, located deep in the axilla near the thoracic wall. During anastomosis, access to the vessels can be difficult in every flap surgery. However, in cases of omental transfer, the vascular pedicle is extremely short and often still situated within the lymphatic or fat tissue of the flap. In the cases in this series, the length of vascular pedicles was between 5 and 10 mm (Video 1). In addition, lymphatic tissue within the omental fat may bulge in front of the vessels, making it even more difficult to perform the anastomosis in a confined space.

**Figure 1:**
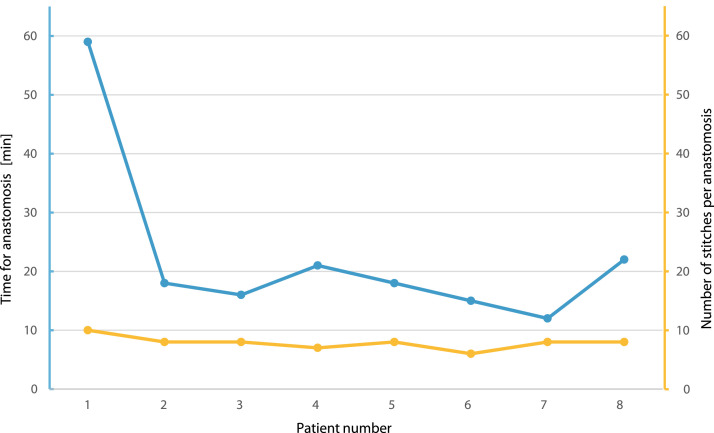
Operating times required for anastomosis (blue line) and number of sutures (orange line) required for each patient.

Anastomotic time was 22.6 ± 26.2 min on average, and 7.9 ± 1.4 stitches were applied in interrupted running single knot technique, which is largely comparable to manual anastomosis (Figure 1). Notably, all anastomoses were patent. The first patient operated in this case series was only the 10^th^ patient overall to be operated with the Symani® Surgical System at our department; therefore, the operating time for anastomosis was remarkably longer (59 min). However, the anastomotic times for the remaining 7 patients were markedly shorter (20.0 ± 2.8 min). Moreover, the operating times for anastomosis using the 3D exoscope were similar to those using a conventional microscope (17.0 ± 7.1 min vs. 16.5 ± 2.1 min).

**Figure 2:**
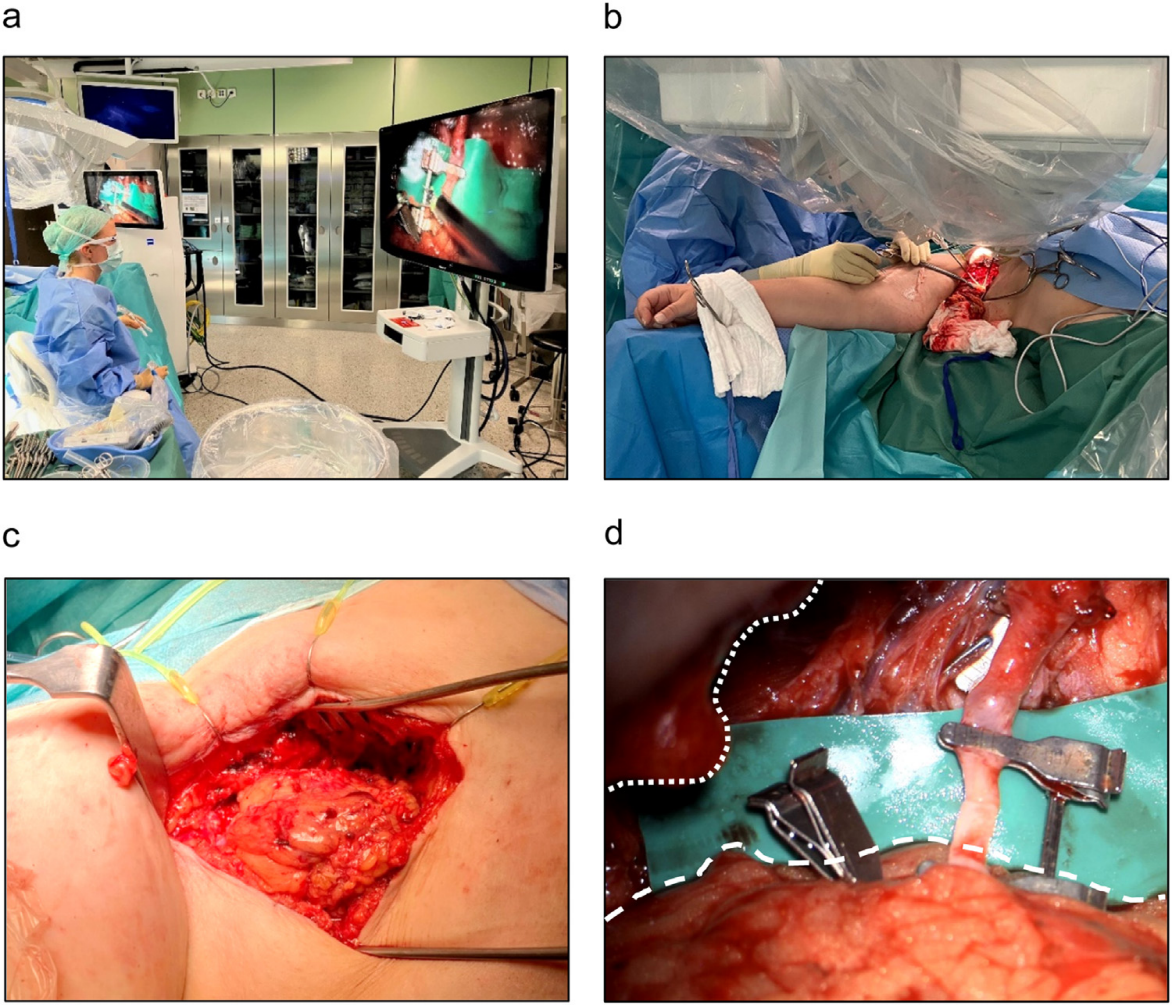
(a) Operative setup in which the surgeon operates the Symani® Surgical System using handheld manipulators and the KINEVO® 900 exoscope using 3D visualization. (b) An assistant can be seen next to the operating field while the robotic arms are in place. (c) Access to the recipient vessel through the axilla with the omental flap. (d) The obstructed view by the entry to the cavity of the axilla (dotted line) and the voluminous tissue transplant (broken line) that is well managed by robotic-assisted surgery is notable.

The surgeon was between the robotic arms when using the conventional microscope. This setup facilitated access to the axilla, making microsurgery easier compared with manual anastomosis, but this did not offer the full benefit of operating from a more comfortable remote position. Therefore, we started using the 3D exoscope as soon as it became available for robotic-assisted microsurgery in this case series (Figure 2a). Based on our personal experience, when using an optical microscope, the resolution and depth perception provided by the attached 3D exoscope are adequate to perform LVA surgery on translucent vessels, which is often the limiting factor of the currently available exoscopes.

With the use of the described setup, the surgeon's positioning could be significantly improved by adding robotic precision to perform anastomosis (Video 2). Moreover, owing to the localization of the axilla, it is usually not possible to have an assistant during microanastomosis to cut the sutures; therefore, the surgeon has to change instruments frequently. The robotic setup with a supermicro dilator and a micro needle holder with cutting function makes the surgeon completely independent, rendering an assistant unnecessary.

In all cases, the flap artery could be accessed well, and anastomoses were performed comfortably despite the very short pedicle in a deep space.

### Case 1

A 50-year-old female patient was suffering from severe secondary lymphedema of the right arm with recurring erysipelas over the last 2 years (Table 1, patient 7). Axillary lymph node dissection was performed before 5 years owing to a metastasized carcinoma of the right breast, followed by adjuvant chemotherapy and radiotherapy (40 Gy). Conservative therapy was performed for 2 years without any beneficial effect. The patient also presented with several perioperative risk factors, including arterial hypertension and an active smoking habit. Harvesting of the omental flap for free lymphatic tissue transfer was complicated by a Roux-Y-gastric bypass performed before 10 years as well as residual obesity of the patient after massive weight loss (body mass index = 42.7 kg/m^2^).

Owing to the high degree of suffering of the patient, VLNT from the omentum to the right axilla was performed. After a successful and uneventful laparoscopic harvest of the omental flap, the right gastroepiploic artery was anastomosed end-to-end with a branch of the right thoracodorsal artery using the Symani® Surgical System in combination with the KINEVO® 900 exoscope. The use of the robot in combination with the KINEVO® 900 exoscope for 3D visualization enabled the surgeon to efficiently perform the anastomosis in the depth of the axilla after extensive scar tissue resection, despite a narrow access to the operating field (Figure 2 a and b, Video 2). In particular, unforeseen factors, such as voluminous lymphatic tissue flap additionally restricting access to the operating field, can be managed well using the robotic surgical system (Figure 2c and d). During the initial follow-up at 2 weeks postoperatively, the patient did not exhibit any signs of complications.

### Case 2

**Figure 3:**
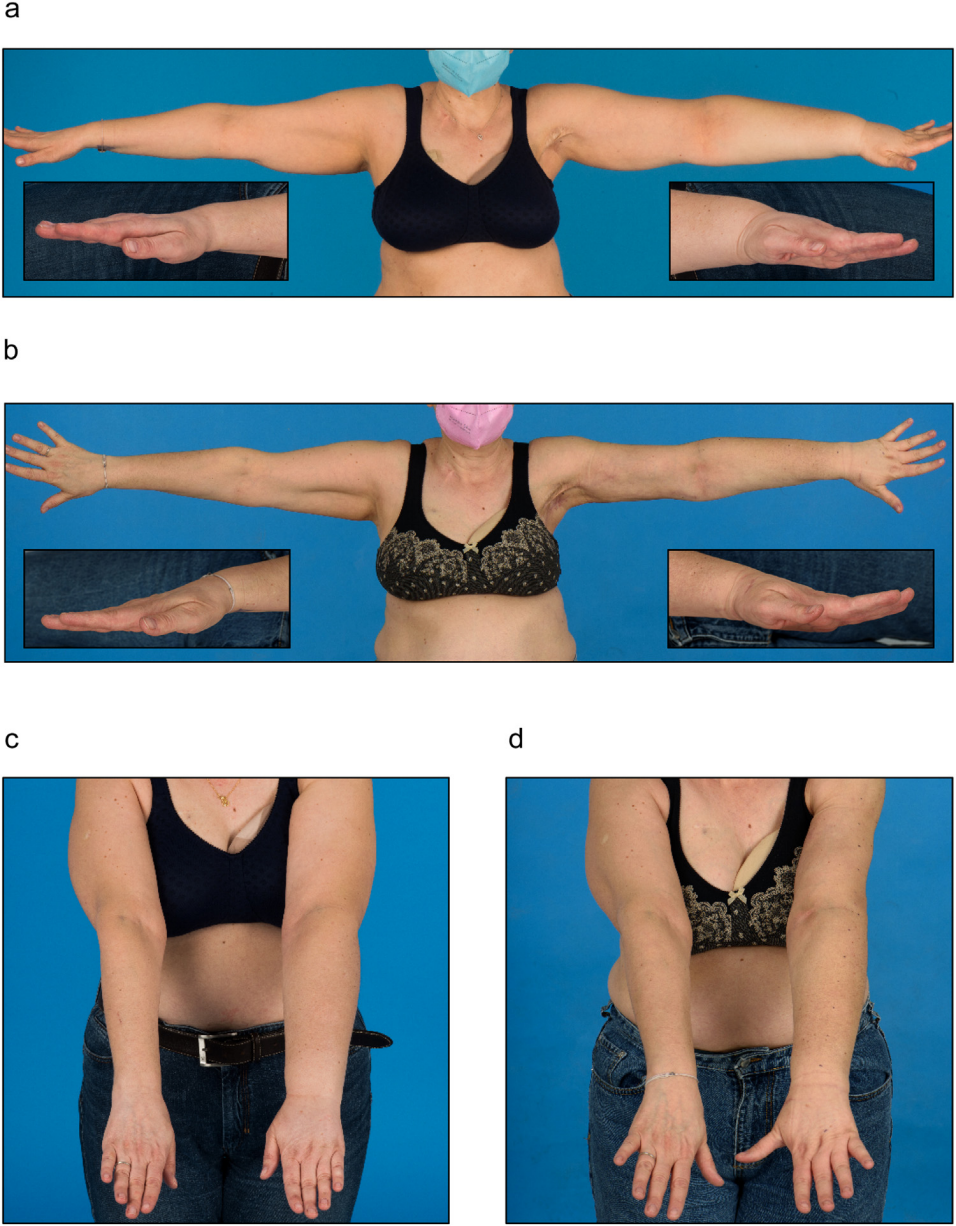
(a and c) Preoperative image showing a significant edema of the left arm. Swelling of the dorsal left hand and forearm is clearly visible compared with the contralateral side (left and right image insets). (b and d) Three months postoperatively, the patient shows markedly reduced edema. Notably, the distal left forearm and hand exhibited visibly decreased swelling compared with the contralateral side (left and right image insets).

Vascularized lymphatic tissue transfer was performed to the axilla in a 51-year-old female patient who presented with secondary lymphedema of the left arm (Figure 3) after mastectomy and axillary dissection before 23 years for breast cancer therapy (see also table 1, patient 4). Adjuvant chemotherapy and radiation therapy were additionally used as part of the oncologic treatment. The patient was suffering from recurrent erysipelas for the past 12 years and needed frequent oral antibiotic treatments. Conservative treatment with pneumatic and sleeve compression and consistent lymphatic drainage did not alleviate the lymphedema. She was offered a combined breast reconstruction and VLNT to the axilla with a deep inferior epigastric artery perforator (DIEP) flap and groin or abdominal wall lymph nodes. However, she did not wish to reconstruct the left breast and opted for the sole treatment of the lymphedema of the left arm, which was very bothersome to her.

**Figure 4:**
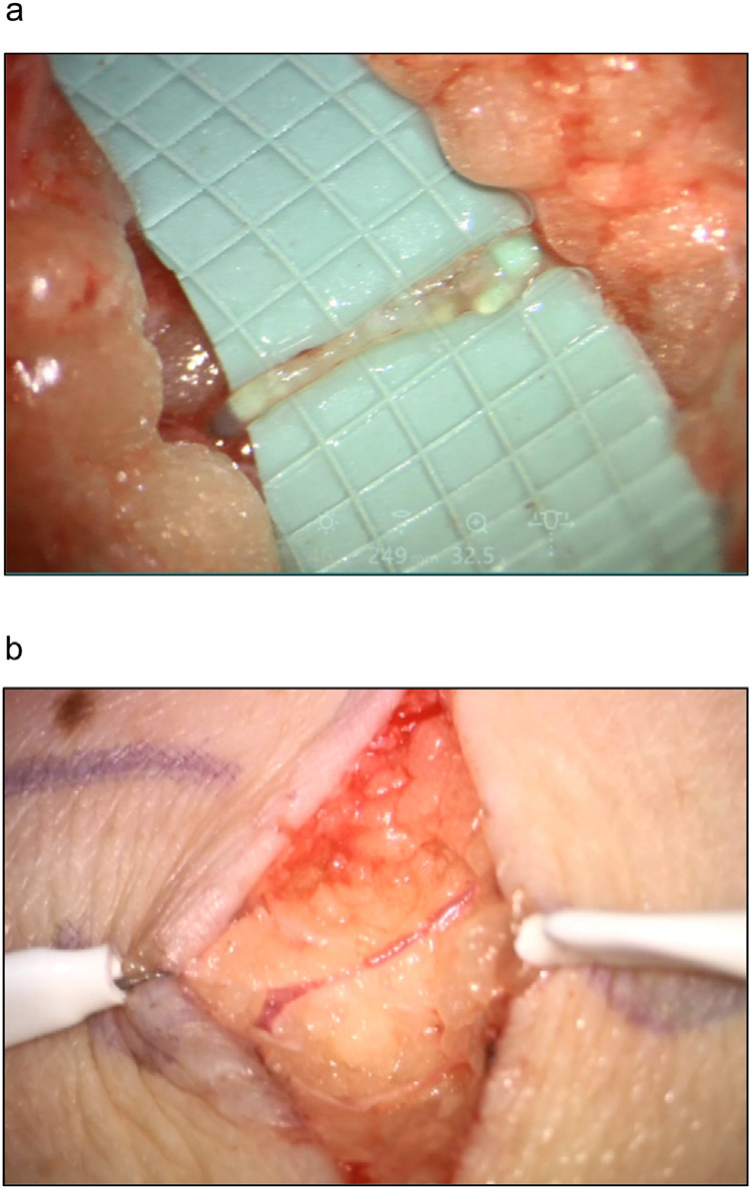
Small lymphatic vessel of 0.3-0.5 mm diameter on the arm of the patient (a) and subcutaneous vein (b) for robotic-assisted lympho-venous anastomosis.

VLNT from the omentum to the left axilla was performed by end-to-end anastomosis of the right gastroepiploic artery with a branch from the left thoracodorsal artery. The Symani® Surgical System was used in combination with the KINEVO® 900 exoscope to perform the arterial anastomosis after extensive scar tissue release within the axilla. Among other features, this microscope is equipped with a robotic visualization platform, a three-dimensional (3D) image display on a 4K monitor that enables operating under exoscopic mode, and intraoperative fluorescence visualization.^10^ In addition to free lymphatic tissue transfer, LVA (Figure 4, Video 3) at the distal left forearm and liposuction of the extremity were performed. At the 3-month follow-up, the patient presented with markedly reduced lymphedema of the left arm. The calculated volume^11^^,^^12^ of the extremity reduced from 3,709 ml preoperatively to 2,773 ml at the 3-month follow-up, corresponding to the decrease of 25.2% and 100% from the extremity and excess volumes, respectively, when compared with the unaffected arm, and the compression sleeve was no longer worn continuously (Figure 3b and d). A significant reduction in swelling of the dorsum of the left hand was noted after VLNT and LVA. The dorsum of the hand is generally the most difficult to treat in arm lymphedema and is not affected by liposuction. Therefore, the result indicates a true postoperative improvement due to reconstructed lymphatic flow, particularly when compression garments are omitted.

## Discussion

In lymphatic reconstructive surgery, the surgeon is required to work on structures in the submillimetric range. This type of surgery has been made possible with the continuous development of supermicrosurgical instruments, high magnification microscopes, and associated technologies (including advanced diagnostic imaging techniques).^13^ However, until the introduction of robotic surgical systems, very little progress was made to directly enhance the surgeon's capabilities. In the last few years, the introduction of robotic surgical systems specifically developed for use in micro- and supermicrosurgeries has shown promising results by replacing traditional manual practice for certain surgical actions and operative steps.

The associated advanced dexterity and superior motion control of robotic surgical systems can reduce tissue trauma and increase the surgeon's precision. Despite these advantages, some important drawbacks remain. To operate on even smaller structures, suture material and visualization systems need to be further developed. The high cost of robotic systems is another limitation. This added expense notably increases the overall operating costs of robot-assisted procedures. For instance, Gundlapalli et al. reported a cost of $16,300 for a robot-assisted breast reconstruction using a DIEP flap compared with $14,800 for a standard DIEP flap reconstruction.^14^ In countries using a diagnosis related group-based compensation system, this cost is not reflected in the hospital compensation, thereby impeding a more widespread use of robotic surgical systems. Moreover, additional expenses include implementation costs (e.g., acquisition and theater modification) and maintenance among others as well as hidden costs, such as those related to longer operating times. The latter is partly attributed to handling of the robot—a skill that must be acquired by the surgeon and nursing staff and can improve over time; however, this could also be partly attributed to the motion scaling technology that may slow the operating speed. Recent studies have shown steep learning curves for the use of surgical systems. In a previous study, our study group demonstrated that the time needed to perform anastomosis using a robotic surgical system decreased consistently after its consistent use, and the time required to perform LVAs was comparable to that required to perform manual anastomoses.^9^ This finding is consistent with the findings of the present study, in which we noticed a steep learning curve with the introduction of the robotic system into our department. However, operating speed may not surpass manual surgical technique owing to motion scaling technology.^15^^,^^16^

Notably, the cost per procedure is volume dependent, indicating that an increased volume of procedures performed using the robotic system reduces the impact of the initial implementation costs per procedure.^17^ Furthermore, increased frequency of use provides a regular opportunity for the surgeon to perfect the handling of the robotic surgical system, which may also benefit the overall operating times and thereby reduce costs in the long run. Therefore, it is sensible to use robotic systems as frequently as possible. However, the surgeon's responsibility of optimizing the use of resources and robotic systems should not be viewed as a default choice. Therefore, we aimed to define certain types of procedures in which the use of a robotic system would provide specific and distinct benefits over manual anastomosis.

The limitations of the study include the fact that all procedures were performed by an experienced microsurgeon who also gained experience in robotic microsurgery in the past 2 years. Because of this fact, the time required to perform an anastomosis surgery in our study may not be comparable to the time required by less experienced microsurgeons. In addition, the setup of the robotic systems currently involves fixed angles in terms of the position of the 2 robotic arms to each other, i.e., the more the arms are lowered, the more the distance between them at the skin level, which may require longer incisions for procedures in deeper regions. However, technical improvements in robotic systems involving flexible arms may resolve this issue in the future.

In conclusion, we found that robot-assisted surgery is particularly beneficial in deep anatomical planes that are difficult to reach and maneuver in manual surgery. If such a deep-seated operating field is intended as a recipient site for a free lymph tissue transfer, robot-assisted surgery should be considered. Although it is difficult to anticipate the precise impact of robotics in microsurgery, we believe that there is an increasing demand for robot-assisted procedures, especially in lymphatic reconstructive surgery. In particular, the central lymphatic system, i.e., the axial lymphatic vessels, including the thoracic duct within the thorax and abdomen, which is located underneath vital organs and situated very dorsally to the vertebral column, may benefit from this procedure. This may be the reason for the little progress in central lymphatic surgery in recent decades, despite a significant increase in peripheral lymphatic surgery of the extremities. However, important advances have been made in understanding the lymphatic system, the pathophysiology of lymphedema, and associated technologies, e.g., imaging of the lymphatic systems (such as intranodal dynamic contrast magnetic resonance lymphangiography). With this technique, the axial lymphatic system can be visualized in great detail, and respective pathologies can be identified. From a surgical perspective, robotic microsurgery is parallel to these developments, allowing surgeons to push the boundaries of reconstruction in anatomic areas previously considered too small and remote to operate, such as the thoracic duct and central lymphatic system.^18^^,^^19^ Ongoing research is required to validate the techniques used in this study in larger clinical settings. Future challenges include cost reduction and technical optimization of robotic systems, which have emerged as one of the most useful and innovative tools in modern plastic surgery.

## Conflict of interest

Nicole Lindenblatt is a consultant and clinical advisor for Medical Microinstruments.

All other authors have no conflict of interest.

## Appendix Supplementary materials

Video 1 Omental flap after completion of arterial anastomosis of approximately 1.5 mm diameter next to the thoracic wall deep within the axilla and 2 vein anastomoses at both ends of the flap (coupler anastomosis). The particularly short pedicle of the flap (5-10 mm) is notable. Image, video 1

Video 2 Arterial anastomosis of the right gastroepiploic artery (1.5 mm) of the omental flap to a branch of the thoracodorsal artery using the Symani® Surgical System. Image, video 2

Video 3 Flow of indocyanine green (ICG) over a robotic-assisted lympho-venous anastomosis of the vessels presented in Figure 4 (approximately 0.7 mm), showing excellent patency without venous backflow. Image, video 3
